# A small effect of conservation agriculture on soil biodiversity that differs between biological kingdoms and geographic locations

**DOI:** 10.1016/j.isci.2021.102280

**Published:** 2021-03-08

**Authors:** Paulina Giraldo-Perez, Victoria Raw, Marc Greven, Matthew R. Goddard

**Affiliations:** 1 The School of Biological Sciences, University of Auckland, Auckland 1010, New Zealand; 2 The School of Life Sciences, University of Lincoln, Lincoln LN6 7DL, UK; 3 The New Zealand Institute for Plant and Food Research Limited – Rangahau Ahumāra Kai, PO Box 845, Blenheim, New Zealand

**Keywords:** Microorganism, Farming Management, Farming Systems, Relation between Agriculture and Environment, Soil Biology, Beverage

## Abstract

Larger easily visible animals and plants are negatively affected by agrochemicals used for intensive food production, but we do not understand the general spatial and temporal effects of agrochemicals on the multitudes of bacteria, fungi, and small invertebrate animals that underpin ecosystem productivity. We sequenced the 16S, ITS2, and COI DNA barcode regions from 648 New Zealand vineyard soil samples managed under either conventional or low-agrochemical-input conservation approaches across two regions and three seasons in 1 year and discovered at least 170,000 phylotypes (taxa) with >97% genetic identity. Management approach correlated with a significant 2%–10% difference in the abundances of phylotypes that differed over regions and seasons. Although the data show that agrochemicals do not have a large effect on soil biodiversity on average, the important finding is that the magnitude of impact differs between taxa types and locations, and some taxa most affected also influence the quality of agricultural produce.

## Highlights

• Agrochemicals have a small effect on soil bacterial, fungal, and animal biodiversity
• The effect differs in space (and less so time) and affects taxa abundances greatest
• There is no effect of agrochemicals on taxa richness
• The size of the effect differs between taxa but is greatest for eukaryotic taxa

## Graphical abstract

**Figure:**
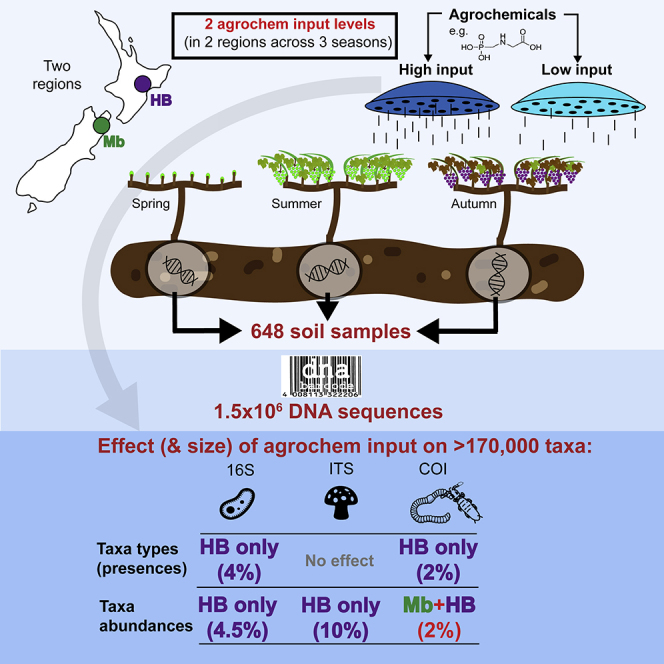

## Introduction

It is well documented that the pesticides used in conventional intensive agriculture decrease the biodiversity of animals and plants that are easily visible to the naked eye (Altieri, 1999; Bengtsson et al., 2005; Billeter et al., 2008; Clough et al., 2005; Gabriel et al., 2010; Gonthier et al., 2014; McLaughlin and Mineau, 1995; Puig-Montserrat et al., 2017; Rundlöf and Smith, 2006), but these taxa represent only a tiny fraction of global biodiversity. Soils harbor one-quarter of the world's biodiversity, and approximately 40% of the globe's land area is dedicated to agriculture. However, the effects of agrochemicals on the vast array of bacteria, fungi, and invertebrate animals, which underpin productivity in ecosystems, particularly via soils, is poorly characterized (Guerra et al., 2020; Harkes et al., 2019; Hartmann et al., 2015; Morrison-Whittle et al., 2017). Greater biodiversity positively correlates with increased ecosystem stability, function, resilience, nutrient recycling, soil detoxification, and pest control, as well as a decreased requirement for fertilizer and pesticide input in agroecosystems (Awasthi et al., 2014; Blanchet et al., 2016; Cardinale et al., 2012; Harrison et al., 2014; McCann, 2000; McGrady-Steed et al., 1997; Médiène et al., 2011; Naeem et al., 1995; Paoletti, 2001; Tilman et al., 2014). There is correspondingly increasing grower and consumer interest in “conservation” agricultural approaches, which consider longer-term productivity and the wider ecological impacts of agriculture: conservation agriculture approaches decrease agrochemical inputs in an attempt to increase biodiversity (Bommarco et al., 2013; de Ponti et al., 2012; Döring et al., 2019; Matson et al., 1997; Tilman et al., 2001). However, most studies to date have only evaluated the effect of different agricultural approaches on larger plants and animals (Bengtsson et al., 2005), but the effect of conservation agriculture on the massively more abundant and important complex micro- and mesofauna communities across time and space is poorly described.

The analysis of bacterial and fungal biodiversity by the amplification and sequencing of millions of 16S and ITS DNA barcodes directly extracted from samples is now commonplace and circumvents problems associated with the fact that >95% of species are missed by culture-based methods (Taylor et al., 2014). The few studies that have used DNA biodiversity estimates to evaluate the effects of agricultural management had limited sampling across locations or time points but have suggested significant biodiversity differences by management approach, but the sizes of effects are relatively small. Hartmann et al. (2015) showed that long-term organic agricultural management at one site had a significant small (∼10%) effect on soil bacterial and fungal communities and conservation management had a significant small (10%) effect on soil fungal biodiversity across multiple New Zealand vineyards in one region at one time point (Morrison-Whittle et al., 2017). Harkes et al. (2019) reported significant small (∼4%) differences between conventional and organic managements from two farms at two time points using bacterial 16S and eukaryote 18S DNA barcodes, and Bonanomi et al. (2016) showed a similar effect in long-term polytunnel systems at one site with the same barcodes (but did not report effect sizes). We are aware of no estimates of the effect of agricultural management on biodiversity using the standard animal COI DNA barcode.

Evidence to evaluate the effects of different agricultural management approaches on broader components of biodiversity, particularly in soils, is lacking, and research that quantifies the impact of commercially relevant agricultural management systems across both time and space will provide evidence to inform policy in this regard (Godfray et al., 2010; Zimmerer and de Haan, 2017). Here we study commercial agricultural sites that operate under either conventional or conservation approaches. We deliberately chose to evaluate a range of commercial sites, rather than experimental plots, to quantify biodiversity differences in authentic agricultural scenarios. We gathered 648 soil samples from 24 New Zealand vineyards located 350 km apart in Marlborough and Hawke's Bay (HB) across spring, summer, and autumn. We tested whether there were multi-kingdom biodiversity differences between management regimes by analyzing bacterial 16S, fungal ITS2, and eukaryote COI DNA barcodes and then went on to estimate the nature and magnitude of any differences and put these into context.

## Results

Analysis of commercial spray diaries (detailed in Table S1) revealed 25% more application events with 3-fold significantly greater input of agrochemical products per hectare in conventional than conservation vineyards across the time period sampled (Mann-Whitney U test, Z = 6.41, n = 574, p = 1.4 × 10^−10^): this substantiates the agricultural management classifications of these sites. One million seven hundred thousand DNA sequence reads were obtained from soils after forward-reverse pairing and quality filtering (561,409 16S; 443,082 ITS2; 724,661 COI). All sequences were clustered into phylotypes of 97% or greater genetic identity, which is a standard level that approximately separates prokaryote species and eukaryote genera (Alberdi et al., 2017; Guerra et al., 2020; Hebert et al., 2003; Konstantinidis and Tiedje, 2005). Twelve COI phylotypes comprising 133,575 reads matched >97% to the *Homo* genus and were removed from all further analyses leaving 172,370 phylotypes and over 1.5 × 10^6^ reads (116,788 16S; 2,557 ITS2; 53,025 COI phylotypes), which to a first approximation estimates the total biodiversity in these ecosystems (Table S2).

It is desirable to attempt to taxonomically classify phylotypes by matching their DNA sequences to those in reference databases. Eighty-five percent of 16S phylotypes were probabilistically assigned to the bacterial kingdom, and 96% of the ITS2 phylotypes were assigned to the fungal kingdom. Actinobacteria was the most abundant bacterial phylum (29%), followed by Proteobacteria (26%), and ascomycetes dominated fungi (79%): a complete breakdown of phylotype abundances at taxonomic levels for 16S and ITS2 barcodes is in Table S2. The identification of COI phylotypes is significantly more challenging as COI databases are far less complete. Comparisons across three different COI databases (see methods) that show 99.99% of phylotypes were assigned to eukaryotes, but only half of these matched to deposits classified at kingdom level with >95% confidence. Approximately half the COI phylotypes matched to deposits assigned to fungi, 3% to animals (metazoans), and ∼0.3% to oomycetes which are fungus-like organisms. Although most eukaryote COI phylotypes we recovered are not yet in databases, this does not prevent their analyses, just their taxonomic assignment.

**Figure 1:**
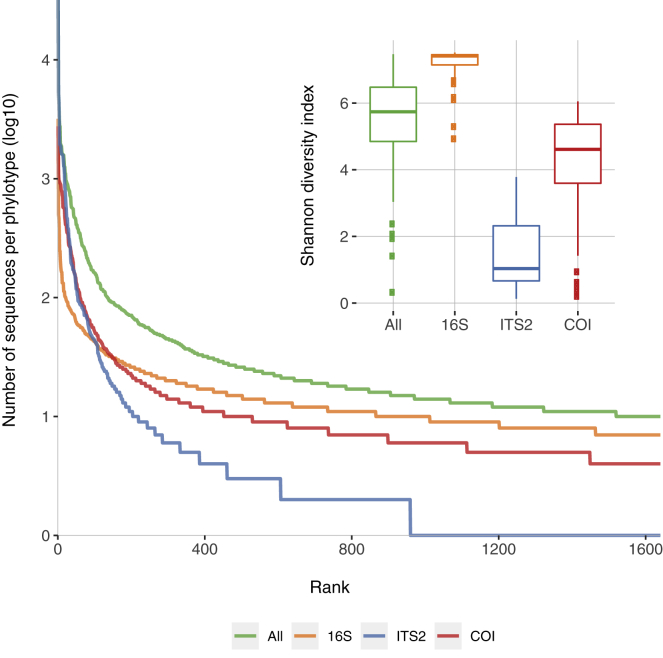
Abundance curves of >97% phylotypes for 16S, ITS, and COI barcodes from 24 New Zealand vineyard soils across spring, summer, and autumn in Marlborough and Hawke's Bay Combined barcode phylotype abundance distribution (all), and by each barcode separately, up to the 1,640th rank, which was the least abundant phylotype. Shannon diversity (inlay) differs between barcodes by Kruskal-Wallis tests (*H* = 168.87, p = 12.23 × 10^−36^), and Dunn's post-hoc tests reveal all distributions differ from one another (p < 0.0007). See also Figure S1.

Eukaryotes contain both ITS and COI regions, but no animals were recovered in the ITS2 data due to the targeting of PCR primers. The 16S barcode thus estimates bacterial biodiversity, the ITS2 estimates fungal biodiversity, and the COI barcode estimates eukaryotic biodiversity generally. We analyzed each barcode separately, and for completeness also combined and analyzed all barcodes. However, combined barcodes will be more heavily influenced by the greater number of 16S phylotypes, and it is possible that some fungal phylotypes overlap between the ITS2 and COI barcodes; we therefore primarily focused on each barcode individually. Rank abundance curves show typical patterns of few common and many rarer phylotypes (Figure 1): bacteria are the richest and most evenly distributed, followed by general eukaryotes and then fungi.

### The effect of management on multi-kingdom biodiversity

The analysis of differential abundances, presences, and counts of phylotypes between management regimes comprises the core of being able to understand whether management approach correlated with differences in biodiversity (Morrison-Whittle et al., 2017). The analyses methods we used were insensitive to differences in data normality, variance, and sample sizes. However, we conservatively sub-sampled (rarefied) to 2,000 DNA sequences per sample for the 16S and ITS2 and 1,000 sequences for the COI barcode to ensure equal sample sizes within each barcode; samples with fewer DNA sequences were removed from analyses. Following rarefaction there were 275,000 DNA sequences and 79,047 phylotypes (Table S3). We tested the null hypothesis that there was no effect of management and went on to evaluate the significance and size of any differences in biodiversity between management approaches when this was rejected. The results for all analyses, including all test statistics, are in Tables S4, S5, S6, and S7, and we only report effect sizes (the proportion of total biodiversity variance explained by a factor) in the text when significant at p < 0.05 for brevity.

### Abundances of phylotypes differ by management and location

**Figure 2:**
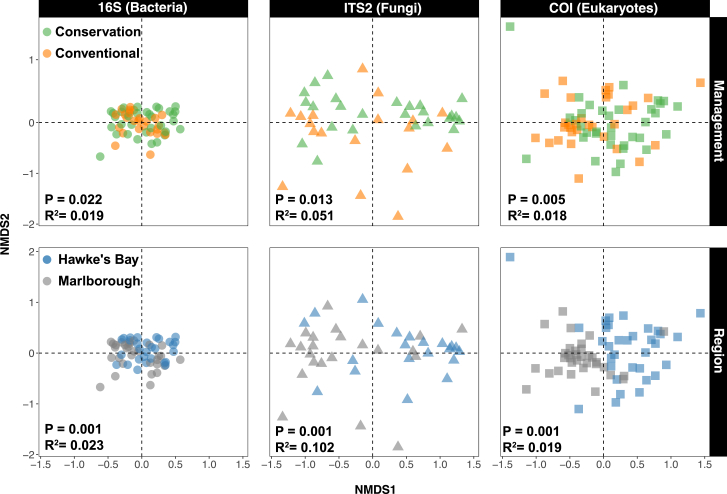
Differences in phylotype abundances Fixed-scale nonmetric multidimensional scaling (NMDS) plots from Jaccard distance matrices representing the difference in abundances of >97% phylotypes by management and region separately from 16S, ITS2, and COI barcodes from the soils of 24 New Zealand vineyards across spring, summer, and autumn in Marlborough and Hawke's Bay. The statistical output from PermANOVA analyses (p values and R^2^) is shown. See also Figure S2.

There was a consistent significant difference in the abundances of all barcodes (and their combination) between management approaches (Figure 2; Table S4). This difference was relatively weaker for 16S and ITS2 (p = 0.022 and 0.013, R^2^ = 0.019 and 0.051), but relatively stronger for differential COI phylotype abundances (p = 0.005, R^2^ = 0.018). There were no significant interactions between management and region for individual barcodes (p = 0.09 to 0.43), but there was a significant difference in phylotype abundances between management approaches for both 16S and ITS2 in HB (p = 0.011 and 0.020, R^2^ = 0.045 and 0.105) but not Marlborough (p = 0.760 and 0.185). There were also differential effects of management between seasons for fungal ITS2 and eukaryote COI phylotypes: both differed by management in summer (p = 0.002, R^2^ = 0.202 and p = 0.028, R^2^ = 0.055 respectively), but there were no differences in bacterial 16S phylotype abundances by season (Table S4).

Phylotype abundances also significantly differed between regions irrespective of management approach (p = 0.001; Table S4; Figure 2). There were also significant differences between phylotype abundances across seasons for 16S and ITS (p = 0.001 and 0.002, mean R^2^ = 0.08), but not for COI barcodes (p = 0.296). Where differential phylotype abundances between management approaches were significant, this had approximately the same effect size as differences by region and season (5.6% for management versus 4.8% and 8.1% for region and season).

### The presences of phylotypes differ by management and location

**Figure 3:**
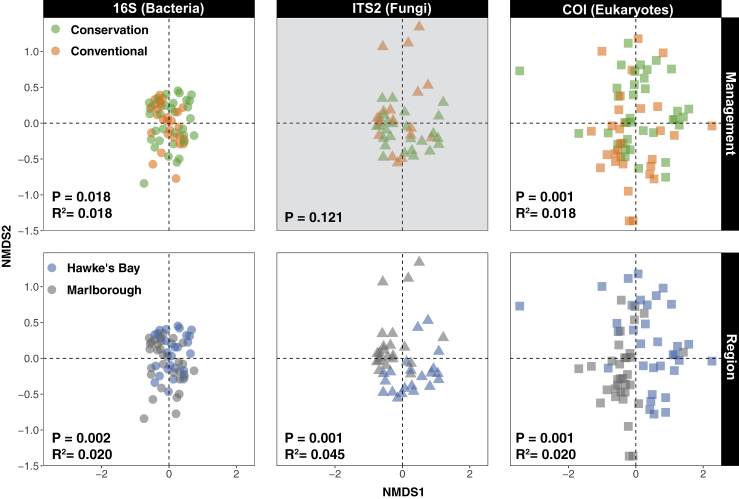
Differences in phylotype presences Fixed-scale NMDS plots from binary Jaccard distance matrices representing the difference in presences of >97% phylotypes by management and region separately for 16S, ITS2, and COI barcodes from the soils of 24 New Zealand vineyards across spring, summer, and autumn in Marlborough and Hawke's Bay. The statistical output from PermANOVA analyses (p values and R^2^) is shown where the differences are significant (at p < 0.05). The grayed-out plot indicates no significant difference (at p > 0.05). See also Figure S3.

There were significant differences between management approaches for the differential presences of 16S and COI phylotypes (p = 0.018, 0.001, Figure 3), and this explained just under 2% of the total variance in phylotype presences across all samples (R^2^ = 0.018; Table S5). However, there were no differences in the presences of fungal ITS2 phylotypes between management approaches (p = 0.121), and no interactions with season and/or region (p > 0.3). There was not a marked significant interaction between management and region or season for the 16S barcode (p > 0.068), whereas there was a significant difference in 16S phylotype presences by management approach in HB (p = 0.005, R^2^ = 0.04) but not Marlborough (p = 0.646), and a weak effect of management in autumn (p = 0.048, R^2^ = 0.054). There were no interactions between management and region or season for the presences of COI eukaryote phylotypes (p > 0.187): the significance and effect size of differences by management were approximately the same across both regions for COI (p = 0.038 and 0.017; R^2^ = 0.034 and 0.033).

Phylotype presences of all barcodes differed significantly between regions irrespective of management approach (p < 0.002 and R^2^ > 0.02; Figure 3; Table S5). The differential presences of bacterial and fungal phylotypes also differed by season (p < 0.004. R^2^ < 0.057), but the COI phylotypes did not (p = 0.11). Where there were significant differences in phylotype presences between management approaches, this explained 2.9% of the total variance in differential phylotype presences across all barcodes, which is almost identical to the size of differences by region (2.8%).

### The numbers of phylotypes mainly differ by location

**Figure 4:**
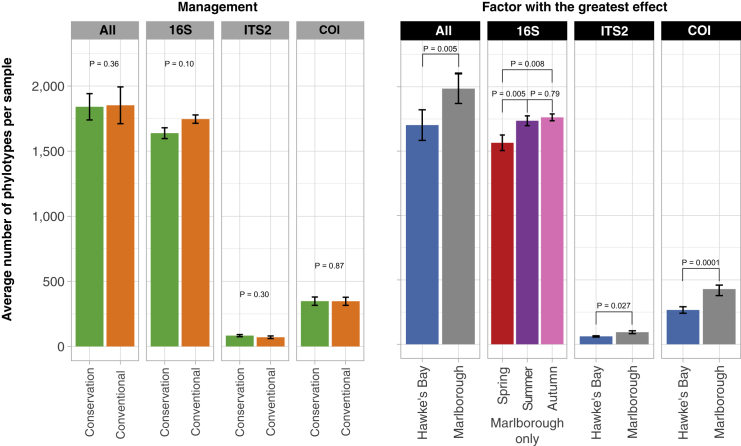
Differences in phylotype counts Mean ± SEM number of >97% phylotypes in soils from all barcodes combined (All) and for each barcode separately, by management (left panel) and by the factor with the greatest effect on phylotype richness (right panel). Kruskal-Wallis tests reveal no significant effect of management on any barcode individually or when combined (p > 0.1), but region and season variously affected phylotype richness. Significant differences revealed by Kruskal-Wallis and Dunn's post-hoc tests (Table S6) are indicated with joined lines above bars with the p values shown. The significant pairwise differences between all three seasons is shown for Marlborough only for the 16S barcode (there was no effect of season on 16S phylotype richness in Hawke's Bay; p = 0.76).

There was no difference in the number (counts) of phylotypes present by management for any barcode individually or when combined (p range 0.1–0.86; Figure 4; Table S6). Although there were weakly significant interactions between management, region, and time for 16S and COI barcodes (p = 0.021 and 0.010 respectively), these did not translate to an effect of management approach in any region or time point separately (p range = 0.09–1). We also analyzed Shannon phylotype diversity indices, which account for the distribution of phylotype counts, and recovered no effect of management (p range 0.11–0.92; Figure S1). Although the above-mentioned analyses are meaningful, they do not evaluate differences in the presences or abundances of specific phylotypes between management approaches as the previous analyses did.

Bacterial phylotypes counts differed most greatly by season in Marlborough (p = 0.003, *E*^2^ = 0.32), but not HB (p = 0.76), where they increased by an average of 297 per sample from budburst to harvest (p = 0.008; Figure 4). Fungal ITS2 and eukaryote COI counts significantly differed by region (p = 0.027 and 0.0001; *E*^*2*^ = 0.116 and 0.23, respectively) and Marlborough had an average of ∼30% more phylotypes per sample (Figure 4).

### Alternative data normalization

We also normalized the entirety of the data with cumulative sum scaling, which is more sensitive to differential sample depths, and analyzed as mentioned earlier (Table S7). This showed the same general patterns as analyses with equal sample depths by rarefaction: no differences in phylotype counts by management and weak to medium significant differences (p ranges 0.03 to 0.001) in presences and abundances of phylotypes between management approaches, with these differences being more apparent in HB than Marlborough for 16S and ITS2, and an average size difference by management that was approximately the same as differences by region.

### Specific phylotype differences by management

Given the significant differential abundances of phylotypes, an indicator analysis was employed to estimate the probability that specific phylotypes had differential proportions between sites managed in different ways. One hundred and forty-six 16S phylotypes significantly differed in abundance by management approach (P_adj_ values range from 0.001 to 0.05): 112 were overrepresented in conventional and 34 in conservation vineyards (Table S8). Thermoleophilia and Rubrobacteria classes were overrepresented in conventionally managed vineyards (p = 0.021), and Bacilli (class) and phylotypes assigned to no lower taxonomic level than Firmicutes (phylum; corrected p = 0.033–0.05) were relatively more abundant in conservation vineyards. Seventeen fungal phylotypes had significantly differential abundances between management approaches: eleven from *Bionectria*, *Malassezia*, *Saccharomyces,* and *Mortierella* genera (corrected p = 0.009–0.037, Table S8) were more abundant in conservation vineyards and six Ascomycete (phylum) phylotypes from *Trichocladium* and unidentified genera are indicative of conventional vineyards. Thirty-four COI phylotypes had statistically greater abundances in conventional and fourteen in conservation vineyards. As there are issues with reliably taxonomically identifying COI phylotypes, we are not able to describe these differences taxonomically (Table S9). However, we attempted to match indicator COI phylotypes to three dedicated COI databases, and we also used nucleotide BLAST searches across the whole of DNA deposits in GenBank (Table S10 and see methods). One of the 14 COI phylotypes overrepresented in conservation vineyards was assigned to *Mus musculus* (mice) with a 99.4% identity, and another nine to *Mus* or Mammalia with 90%–96% identities, meaning these are highly likely from mammals, probably Muridae (rodents). Two other COI phylotypes that were relatively more abundant in conservation sites have ∼97% matches to Ascomycete fungi, but any finer robust taxonomic classification is not possible. The last COI conservation-indicative phylotype has not yet been described as the best matches in GenBank are ∼94% to both Hemiptera (insect, tree bugs) and Ascomycetes (fungi). All 34 conventional indicative COI phylotypes have poor matches (83%–95%) to various Ascomycete species, and it seems these fungal species are yet to be added to databases. We detected DNA sequences in the raw non-rarefied data assigned to *Botryosphaeria spp., Eutypa lata*, and *Phaeomoniella spp.,* fungal pathogens that are implicated in canker and esca-like vine trunk diseases, but there was no significant difference in the abundances of these disease agents between management approaches (corrected p > 0.072). No fungal phylotypes matching to *Erysiphe, Plasmopara,* or the *Botrytis* genera, which contain species that cause grape powdery and downy mildew and bunch rot, were recovered, but recall that these are soil samples.

## Discussion

Overall there was a detectable difference in soil-derived DNA estimates of multi-kingdom biodiversity that correlated with the way these sites had been agriculturally managed (Table 1), but the sizes of biodiversity differences were generally small (<10%). However, the effect of different agricultural approaches on biodiversity across these sites differed between bacterial, fungal, and eukaryote phylotypes and was contingent on location (and season to a lesser extent). The abundances and types of eukaryote COI phylotypes showed the strongest and most consistent differences between management approaches irrespective of region, but with a suggestion of a greater difference in summer. However, there were only differences in fungal and bacterial biodiversity between agricultural management approaches in HB, but not in Marlborough (Table 1), with a signal for greater differences in fungal phylotype abundances by management in summer.

**Table 1:** The differential effects of conservation and conventional agricultural management on numbers, types, and abundances of 16S, ITS2, and COI >97% phylotypes from 24 New Zealand vineyard soils across spring, summer, and autumn in Marlborough and Hawke's Bay (HB)

	Number	Type	Abundance
**16S**	0.100	HB only - **0.005** R^2^ = 0.04	HB only - **0.011** R^2^ = 0.045
**ITS2**	0.301	0.121	HB only - **0.020** R^2^ = 0.105
**COI**	0.867	**0.001** R^2^ = 0.018	**0.005** R^2^ = 0.019

p values are shown and derive from Kruskal-Wallis (number of phylotypes), and PERMANOVA (types and abundance) analyses and HB-specific effects are indicated where they are present and indicate that there was no effect of management on that component of biodiversity in Marlborough. The effect size (R^2^) is included where there was a significant effect of management (at p < 0.05, in bold).

Although a significant effect of management approach on biodiversity was revealed overall, this was not always manifest at each individual point. This may be because the overall effect is manifest due to smaller cumulative spatiotemporal differences by management and/or may be a signal for a differential effect of agrochemicals on biodiversity across time and space. In line with this differential effect idea, the observations of differences in bacterial and fungal communities between HB and Marlborough (which are 350 km apart) irrespective of management approach shown here agrees with previous data from these and other New Zealand and global regions showing such patterns (Bokulich et al., 2014; Morrison-Whittle and Goddard, 2015; Taylor et al., 2014). This suggests that region-specific bacterial and fungal communities are differentially affected by agrochemicals. Our findings are in line with previous work evaluating fewer taxa/barcodes, regions, and time points (Hartmann et al., 2015; Morrison-Whittle et al., 2017; Harkes et al., 2019), including inferences of a greater effect of agrochemicals on metazoa (Bonanomi et al., 2016) , and extend these to show that agricultural management approach affects different bacterial, fungal, and animal taxa in different regions at different times of the year in different ways. This finding echoes patterns seen for the effects of pesticides on specific larger animal and plant taxa (Bengtsson et al., 2005; Gabriel et al., 2010). Although we have measured multiple time points in one year, the extent to which these patterns hold across multiple years with changing climates is of interest. Here we report the first year of a 5-year study, and subsequent analyses will reveal how any effect of management changes across greater time periods.

To put the size of the effects of agricultural management approach on biodiversity into context, this explained 2%–10% of the variance in abundances of the ∼170,000 trans-kingdom phylotypes identified across these sites, and this is on the same order of magnitude as has been estimated in other agricultural soils at fewer places and time points (Hartmann et al., 2015; Morrison-Whittle et al., 2017; Harkes et al., 2019). This may not seem large on first inspection, but the biodiversity variance in most ecosystems is large, particularly for microbial diversity, and it is therefore noteworthy that differences by management approach were detected at all. Furthermore, the magnitude of differences in biodiversity between management approaches is approximately the same as the size of differences in biodiversity across hundreds of kilometers and seasons within a year in a temperate climate. Thus, choice of agricultural management approach can subtly but significantly alter soil biodiversity to the same extent as that imposed by different regions and seasons in many countries.

Soils not only have huge levels of biodiversity but also serve as a repository for DNA from many organisms in an ecosystem (Drummond et al., 2015; Taberlet et al., 2012), and this DNA may have derived from whole or parts-of and live and recently dead organisms. The biodiversity metrics used here do not account for the ecological function of phylotypes or communities, meaning we cannot comment on how the subtle changes in biodiversity translate into changes in ecological function. The inference of increased rodent abundance in conservation vineyards may be a result of more subtle changes in abundances or levels of toxicity accumulation in organisms on which they feed (Fremlin et al., 2020), or changes in structural habitats due to differential ground plants. *Saccharomyces* (single-celled fungi) phylotypes had greater prevalence in conservation vineyards. Although rare in the environment generally, *Saccharomyces* are found in soils and also are keystone species in fruit nutrient turnover and the fermentation of food and beverages (Goddard, 2008; Knight and Goddard, 2015). Populations of *Saccharomyces* are regionally genetically differentiated in New Zealand, including the regions sampled here (Knight and Goddard, 2015), and globally (Peter et al., 2018; Almeida et al., 2014). Work also shows these regionally distinct yeast populations impart small but significantly different sensorial differences via secondary metabolites during fermentation, which in turn contribute to a wine's distinctness, quality, and value (Knight et al., 2015). This suggests that the way agroecosystems are managed may also alter components of biodiversity that contribute to the nature/quality of agricultural produce. Overall, this study provides evidence that different commercially authentic agroecosystem management approaches that manipulate the levels of agrochemical inputs have a measurable but subtle effect on biodiversity, but the nature of this effect differs between organisms and across time and space. The next challenge is to understand what drives the differential effects of agrochemicals on different aspects of soil biodiversity across time and space.

### Limitations of the study

The main question addressed by this study is the overarching effect of agrochemical inputs on bacterial, fungal, and general eukaryote biodiversity. The main limitations are noted in the discussion but are listed here again for completeness. The first possible limitation of the study is the extent to which these observations translate to other agricultural systems in other locations, although these findings are in line with those of other studies in other systems at other places and times. Another potential limitation is the use of soil-derived DNA to estimate biodiversity: some DNA may have derived from dead organisms and eDNA from larger animals may be relatively under-represented. The final potential limitation is that this study evaluates biodiversity but not community function.

### Resources availability

#### Lead contact

Further information and requests for resources and reagents should be directed to and will be fulfilled by the lead contact: Matthew Goddard (mgoddard@lincoln.ac.uk).

#### Materials availability

This study did not generate new reagents.

#### Data and code availability

Raw sequence data are available at GenBank: PRJNA635690, and the published article contains tables derived from the raw sequence data.

## Methods

All methods can be found in the accompanying transparent methods supplemental file.

## Supplemental information

Document S1. Transparent methods and figures S1–S3

Table S1. Commercial spray diary data broken down by management and product type, related to Figures 2, 3, and 4

Table S2. Raw 16S, ITS2, and COI phylotype tables, in separate tabs, with taxonomic assignments where available, related to Figures 1–4 and Table 1

Table S3. Rarefied 16S, ITS2, and COI phylotype tables, in separate tabs, with taxonomic assignments where available, related to Figures 1–4 and Table 1

Table S4. PERMANOVA results on the differential abundances of phylotypes, related to Figure 2

Table S5. PERMANOVA results on the differential presences of phylotypes, related to Figure 3

Table S6. Kruskal-Wallis test results on the differential numbers of phylotypes, related to Figure 4

Table S7. Biodiversity analyses based on phylotype tables normalized via Cumulative Sum Scaling (CSS), related to Table 1

Table S8. 16S and ITS indicator phylotypes, related to Figure 2

Table S9. Comparison of COI phylotype taxonomic assignments, related to Figures 1–4

Table S10. COI indicator phylotype, related to Figure 2
