# Supplementary material for: A small effect of conservation agriculture on soil biodiversity that differs between biological kingdoms and geographic locations
Source: iScience. 2021 Mar 8;24(4):102280. doi: 10.1016/j.isci.2021.102280 (PMC8008184; doi:10.1016/j.isci.2021.102280)
Supplement: Document S1. Transparent methods and figures S1–S3 [file mmc1.pdf]

iScience, Volume *24*

Supplemental information

A small effect of conservation agriculture on soil biodiversity that differs between biological kingdoms and geographic locations

Paulina Giraldo-Perez, Victoria Raw, Marc Greven, and Matthew R. Goddard

Supplemental Figures.

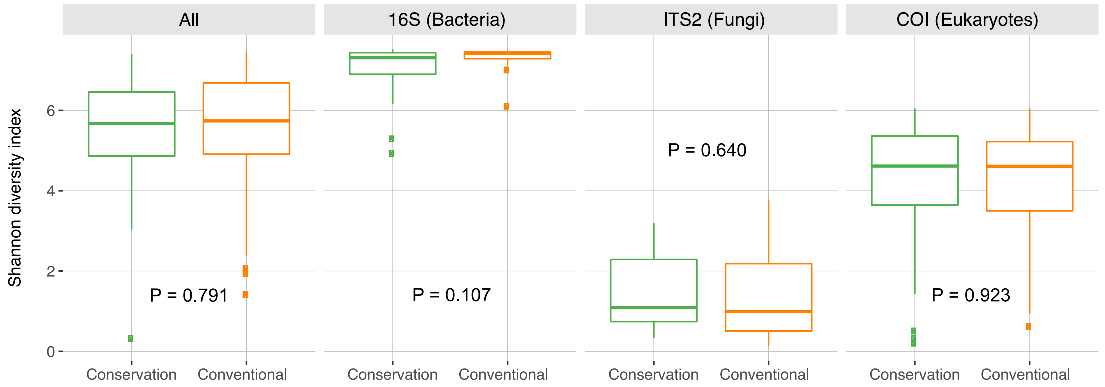

Figure S1: Shannon indices by management and barcode, with P values from analyses with Kruskal-Wallis tests, related to Figure 1.

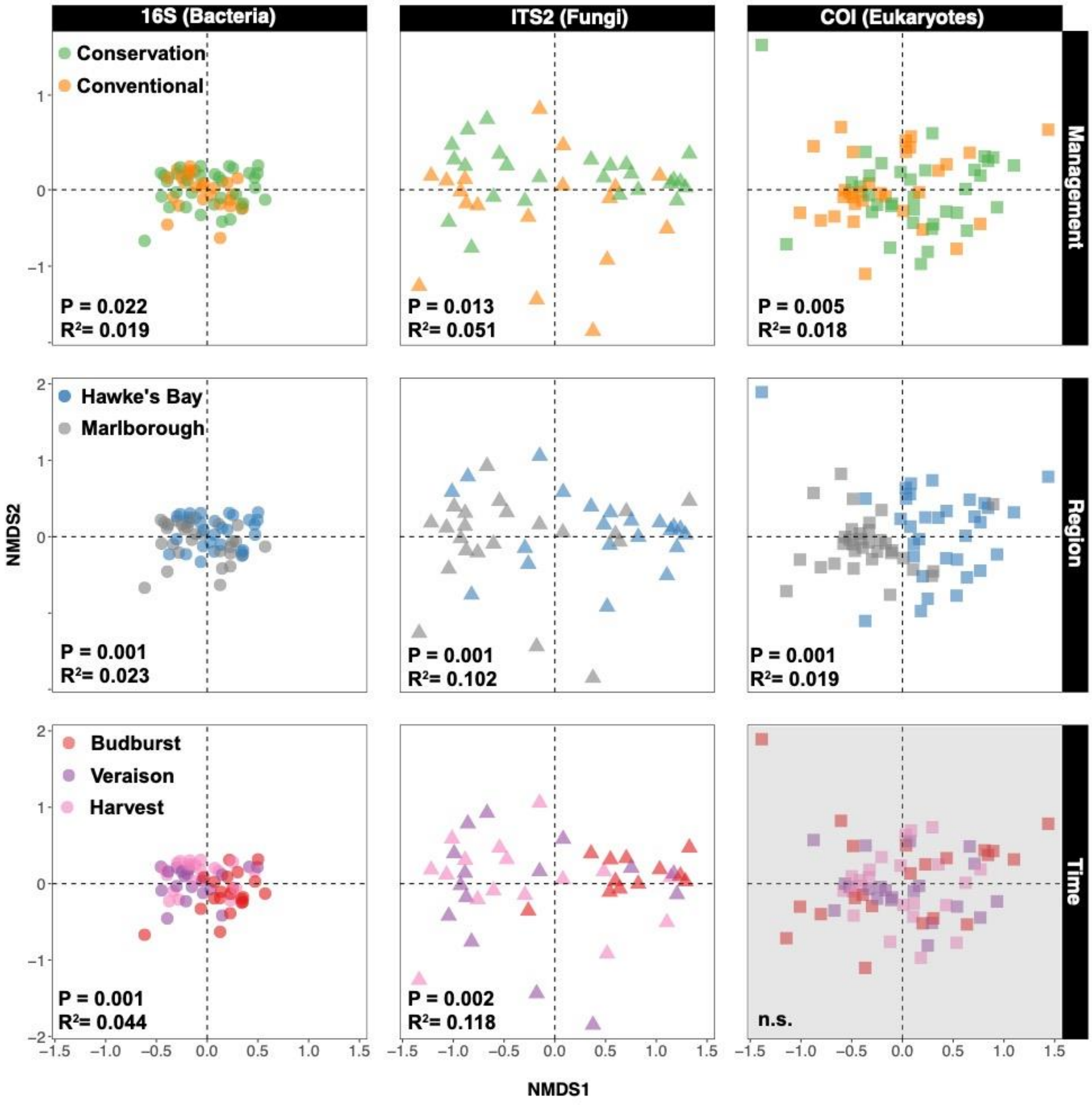

Figure S2: Fixed-scale NMDS abundance based Jaccard distance plots for >97% phylotypes from 24 New Zealand vineyard soils with PermANOVA P and R^2 values where the effects are significant (at P<0.05), related to Figure 2.

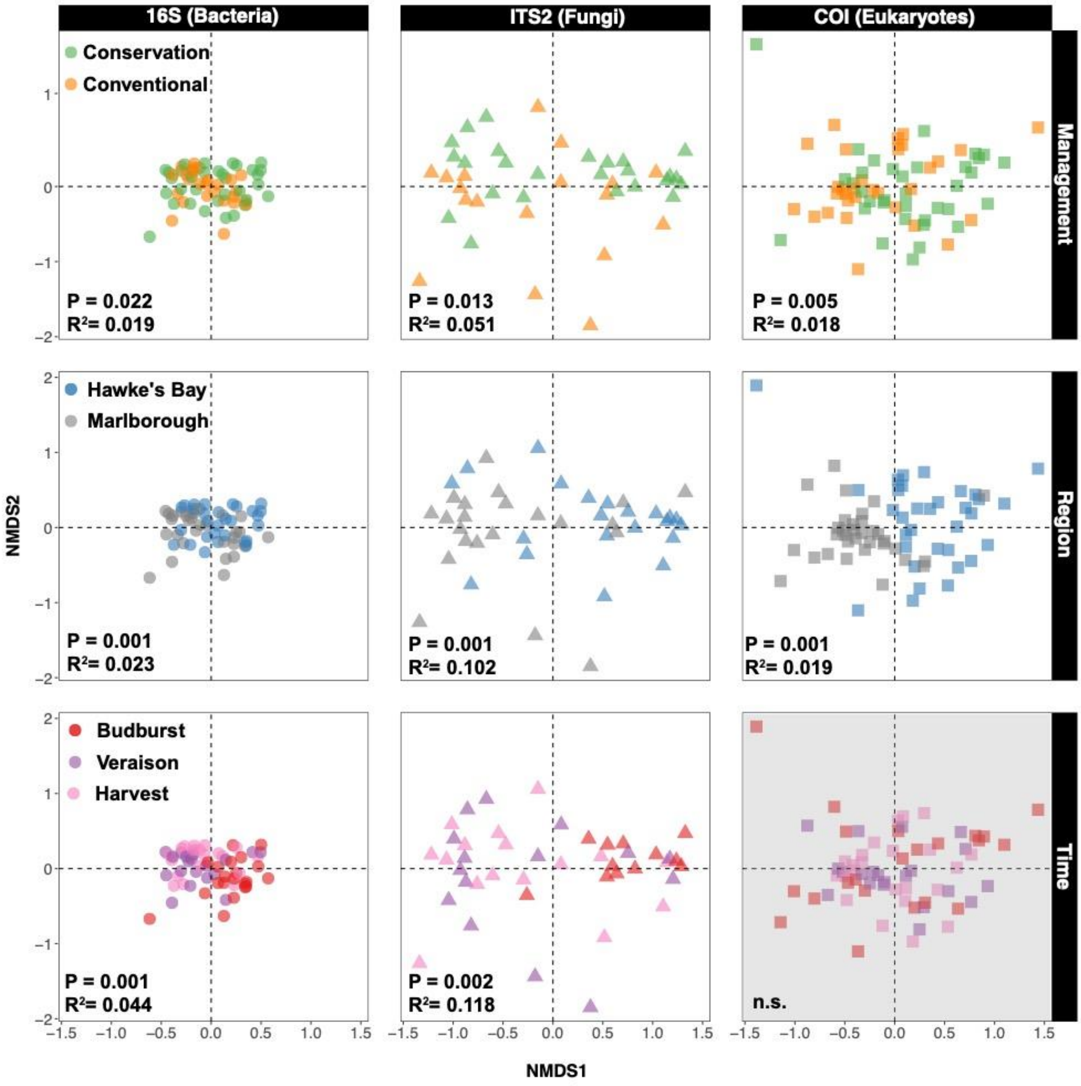

Figure S3: Fixed-scale NMDS binary based Jaccard distance plots for >97% phylotypes from 24 New Zealand vineyard soils with PermANOVA P and R^2 values where the effects are significant (at P<0.05), related to Figure 3.

Transparent Methods

Sites and Sampling

Soil was sampled from 24 commercial *Vitis vinifera* vineyards across two major wine-producing regions of New Zealand approximately 350km apart: Hawke's Bay (HB; 39° S, 177° E) and Marlborough (Mb; 41.5° S, 174° E). Thirteen vineyards were classified as conservation (HB=7, Mb=6) and 11 as conventional (HB=5, Mb=6). Three grape varieties were planted across these vineyards: 12 Sauvignon blanc (6 in each region), 6 Merlot (HB only) and 6 Pinot Noir (Mb only). All vineyards are audited and accredited under the Sustainable Winegrowing NZ programme or are BioGro NZ certified, which ensures growers undertake international sustainability 'best practice' for minimal impact on the environment. Conservation agriculturally managed vineyards attempted to minimise synthetic chemical inputs and gave preference to other means of weed (e.g. under-vine cultivation and mowing) and pest control, or vine nutritional deficiencies. Soil was collected at three time-points: spring (budburst: late October 2015, recall NZ is in the Southern hemisphere), summer (véraison: mid-February 2016) and autumn (harvest: late April 2016). Samples were collected from a random angle and distance in a 16m-radius from each of nine predefined dispersed loci within each vineyard and comprised 20 cm-deep soil cores. Four hundred grams (200g from the under-vine area, and 200g in the inter-row

area) were combined at each locus. A total of 216 samples per time point were collected, comprising 648 samples across the entire study. All tools were sterilised with Trigene® (1:20) and 70% ethanol and rinsed with sterile water prior to collection. Samples were placed directly into sterile containers and kept at 4°C immediately after collection and whilst under transport to the laboratory.

Soil processing
Upon arrival at the laboratory, the nine samples from each vineyard were thoroughly mixed in equal parts and frozen at -80°C for at least 2 months. Samples were allowed to thaw at room temperature, then oven-dried at 65°C until reaching constant weight after approximately 3 days, then homogenised by sifting through a sterile 2mm-sieve, and stored again at -80°C.

DNA extraction and barcode amplification
DNA was extracted from 250mg of soil in triplicate with Zymo Research Soil Microbe DNA Kit™ following the manufacturer's instructions, but with two additions: in Step 1 samples were incubated for 10min at 55°C prior to bead beating; at Step 10 samples were incubated for 5 minutes at 55°C before centrifugation. The triplicate DNA extractions from each sample were pooled. The bacterial V3-V4 16S ribosomal domain was amplified using the primers Bakt_341F: 5'-CCTACGGGNGGCWGCAG-3 and Bakt_805R: 5'-GACTACHVGGGTATCTAATCC-3' (Herlemann et al., 2011). Fungal Internal Transcribed Spacer 2 (ITS2) regions were amplified using the primers ITS3: 5'-GCATCGATGAAGAACGCAGC-3' and ITS4: 5'-TCCTCCGCTTATTGATATGC-3' (White et al., 1990). The eukaryote Cytochrome Oxidase subunit I gene (COI) was amplified with primers mlCOIintF: 5'-GGWACWGGWTGAACWGTWTAYCCYCC-3' (Folmer et al., 1994) and jgHCO2198R: 5'-TAIACYTCIGGRTGICCRAARAAYCA-3' (Geller et al., 2013). All primers included Illumina adapter sequences and amplicons were generated with KAPA HiFi HotStart ReadyMix DNA polymerase (Roche), cleaned using AMPure XP (Agencourt) and quality-checked using Qubit® dsDNA HS Assay kit and Agilent 2100 Bioanalyzer system. Negative controls were included in all PCR batches and none showed contamination. Amplicons were pooled in equimolar concentrations for library construction, and all 216 amplicons (24 vineyards x 3 time-points x 3 barcodes) sequenced in one run on an Illumina MiSeq™ with 2x300bp chemistry at the University of Auckland.

Bioinformatics
Reads were differentiated into kingdoms with fastq-multx (Aronesty, 2011) using primer sequences as identifiers; all unassigned sequences were removed. Data from each barcode were analysed independently. After forward/reverse pairing and low-quality filtering (phred score set to Q20), chimeras were removed and analysed with QIIME (Caporaso et al., 2010). USEARCH (Edgar, 2010) was employed to determine phylotypes that clustered with at least 97% identity: this cut-off approximates differences between phylotypes generally (Alberdi et al., 2017; Guerra et al., 2020; Hebert et al., 2003; Konstantinidis et al., 2005). All phylotypes comprising a single read (singletons) were removed. The accurate taxonomic assignment of any phylotype is contingent on that type having been described, and the corresponding barcode DNA sequence obtained and deposited in a database. Such databases are most comprehensive for 16S (bacteria), somewhat comprehensive for ITS2 (fungi), but are far less comprehensive for COI (general eukaryotes) barcodes. In the absence of exact matches, one can attempt to probabilistically assign phylotypes to various taxonomic levels based the extent to which it matches known taxa, which is again contingent upon DNA database completeness. The taxonomic identities of 16S and ITS2 phylotypes were determined by taking the representative sequence for each and comparing to the appropriate reference database: SILVA v123 (Pruesse et al., 2007) for 16S; UNITE v7.1 (Kõljalg et al., 2013) for ITS2. The RDP Classifier 2.2 (Wang et al., 2007) was used to identify 16S sequences, and BLAST (Altschul et al., 1990) for ITS2. The reference databases for COI barcode sequences are far less comprehensive than those for 16S and ITS. This means the taxonomic assignment of COI phylotypes representative sequences is still extremely unreliable. While several good attempts have been made to curate COI databases, we found these to only be able to consistently taxonomically identify ~50% of COI phylotypes we recovered after cross comparisons of taxonomic identifications from BOLD (Ratnasingham et al., 2007), Midori (Leray et al., 2018) and terrimporter (Porter and Hajibabaei, 2018); we note Midori only considers metazoan taxa; Table S9 shows this cross comparison. We used BOLD and Midori RPD taxonomic assignment

followed by manual BLAST searches to identify and remove 12 phylotypes that matched >97% to human COI deposits in Genbank. This is conservative as the average genetic variance in the global human population is ~0.1%, which equates to less than 1bp difference across the ~330bp COI barcode amplified, whereas the >97% threshold we employed translates to a 10bp difference across the COI barcode sequence. Only 3 phylotypes matched 100% to *Homo sapiens* deposits in Genbank. We analysed 53,037 non-human >97% COI phylotypes as an estimate of the biodiversity of eukaryotes generally. We appreciate there will have been primer bias, and while the COI barcode primers we employed were designed to principally target metazoan invertebrate arthropods, annelids, nematodes and other worms, and any vertebrate DNA present, they may well have also amplified a range of fungi, amoeba and oomycetes as well. We focused on taxonomically identifying those COI phylotypes that prove to significantly differ between vineyards and used the same BOLD, Midori and terrimporter (adapted for use in QIIME by customising the gb2qiime.py script from https://bitbucket.org/beroe/mbari-public) and manual BLAST searches to estimate the taxonomic identity of these COI phylotypes (Table S10).

Data analysis
As for any ecological sampling effort, samples with greater DNA sequence sample depth will tend to recover more phylotypes, and this was the case with our data: there was a significant positive correlation between 16S read depth and numbers of phylotypes recovered (Pearson r = 0.92, P<0.0001). Given a few samples had very low sequences (<1,000) and the large variance in DNA read number between samples of the same barcode of ~2 orders of magnitude (Table S2), then equal sample efforts across samples by equal sub-sampling (rarefaction) was important to ensure the hypothesis was fairly tested. 16S and ITS2 data were rarefied to 2,000 reads, and COI data to 1,000 reads per sample, and samples with fewer than these reads were discarded. The final rarefied phylotype table and number of samples that were included for rarefied analyses are shown in Table S3. The process of rarefying excludes rarer phylotypes and samples with low sequence numbers and so we also normalised phylotype read tables using a Cumulative Sum Scaling method. Different normalisation techniques have been developed in order to address clustering patterns and variability in phylotype abundance data such as those derived from meta-barcode sequences. The nature of our data, hypotheses and preference for a conservative approach best align with rarefying (Weiss et al., 2017).

Community distributions. Count distributions were visualised using relative abundance versus abundance rank (Whittaker plots), and statistically analysed with Kruskal-Wallis analysis of variance, and Dunn's post-hoc test of Shannon Diversity indices.

Biodiversity metrics. Each biodiversity metric (numbers, types and abundances of phylotypes) was analysed with the following levels: management: 2 levels (conservation and conventional); region: 2 levels (Hawke's Bay and Marlborough); and time: 3 levels (spring, summer, autumn). Kruskal-Wallis tests were used to analyse differences in phylotype numbers as the data were not normally distributed; P values were calculated by comparing each value of H to the appropriate $\chi^2_{[a-1]}$ distribution, where a = number of groups, and epsilon-squared estimates of effect size were calculated with $E^2 = H/((n^2-1)/(n+1))$, where n = number of observations (Tomczak and Tomczak, 2014). We also analysed Shannon Diversity indices and post-hoc Dunn's tests (Dunn 1964). Non-parametric permutational multi-way multivariate ANOVA (PERMANOVA) (Anderson, 2017), which does not require data to be normally distributed, were conducted to analyse types and abundances of phylotype on binary (presence/absence) and abundance based Jaccard dissimilarity matrices, with 100,000 permutations, and effect sizes indicated by R^2 (note R^2 are only available for main effects, but not interactions between them). Non-parametric tests and multifactorial analyses of variance were employed to evaluate the effect of management on specific pathogens on non-rarefied data. All factors were treated as fixed effect variables, and construction of dissimilarity matrices, statistical analyses and plots, including NDMS, were conducted in QIIME and R (R Core Team, 2020) with the packages 'phyloseq' (McMurdie and Holmes, 2013), 'vegan' (Oksanen et al., 2020). Indicator phylotype were determined using the 'indval' function of the R package 'labdsv' (Roberts, 2019).

Supplemental References (references that are already included in the main body of the paper are not listed again)

Altschul, S. F., Gish, W., Miller, W., Myers, E. W. and Lipman, D. J. (1990). Basic local alignment search tool. J. Mol. Biol. 215, 403–410.

Anderson, M. J. (2017). Permutational Multivariate Analysis of Variance (PERMANOVA). Wiley StatsRef: Statistics Reference Online.

Aronesty, E. (2011). ea-utils :Command-line tools for processing biological sequencing data; http://code.google.com/p/ea-utils

Caporaso, J. G., Kuczynski, J., Stombaugh, J., Bittinger, K., Bushman, F. D., Costello, E. K., Fierer, N., Peña, A. G., Goodrich, J. K., Gordon, J. I. et al. (2010). QIIME allows analysis of high-throughput community sequencing data. Nat Meth 7, 335–336.

Dunn, O. J. (1964). Multiple Comparisons Using Rank Sums. Technometrics 6, 241–252.

Edgar, R. C. (2010). Search and clustering orders of magnitude faster than BLAST. Bioinformatics 26, 2460–2461.

Folmer, O., Black, M., Hoeh, W., Lutz, R. and Vrijenhoek, R. (1994). DNA primers for amplification of mitochondrial cytochrome c oxidase subunit I from diverse metazoan invertebrates. Mol. Mar. Biol. Biotechnol. 3, 294–299.

Geller, J., Meyer, C., Parker, M. and Hawk, H. (2013). Redesign of PCR primers for mitochondrial cytochrome c oxidase subunit I for marine invertebrates and application in all-taxa biotic surveys. Mol. Ecol. Resour. 13, 851–861.

Herlemann, D. P., Labrenz, M., Jürgens, K., Bertilsson, S., Waniek, J. J. and Andersson, A., F. (2011). Transitions in bacterial communities along the 2000 km salinity gradient of the Baltic Sea. ISME J. 5, 1571–1579.

Kõljalg, U., Nilsson, R. H., Abarenkov, K., Tedersoo, L., Taylor, A. F. S., Bahram, M., Bates, S., Bruns, T. D., Bengtsson-Palme, J., Callaghan, T. M., et al. (2013). Towards a unified paradigm for sequence-based identification of fungi. Mol. Ecol. 22, 5271–5277.

Leray, M., Ho, S-L., Lin, I-J. and Machida, R. J. (2018). MIDORI server: a webserver for taxonomic assignment of unknown metazoan mitochondrial-encoded sequences using a curated database. Bioinformatics 34(21), 3753-3754.

McMurdie, P. J. & Holmes, S. (2013). phyloseq: An R Package for Reproducible Interactive Analysis and Graphics of Microbiome Census Data. PLoS ONE 8, e61217.

Oksanen, J, Blanchet, F. G., Friendly, M., Kindt, R., Legendre, P., McGlinn, D., Minchin, P. R., O'Hara, R. B., Simpson, G. L., Solymos, P., et al. (2020). vegan: Community Ecology Package. R package version 2.5-7. https://CRAN.R-project.org/package=vegan

Porter, T. M. and Hajibabaei, M. (2018) Automated high throughput eukaryote CO1 metabarcode classification. Scientific Reports, 8, 4226.

Pruesse, E., Quast, C., Knittel, K., Fuchs, B. M., Ludwig, W., Peplies, J. and Glöckner, F. O. (2007). SILVA: a comprehensive online resource for quality checked and aligned ribosomal RNA sequence data compatible with ARB. Nucleic Acids Res. 35, 7188–7196.

R Core Team (2020). R: A language and environment for statistical computing. R Foundation for Statistical Computing, Vienna, Austria. URL https://www.R-project.org/.

Ratnasingham, S. and Hebert, P. D. N. (2007). BOLD: The Barcode of Life Data System (http://www.barcodinglife.org). Mol. Ecol. Notes 7, 355–364.

Roberts, D. W. (2019). labdsv: Ordination and Multivariate Analysis for Ecology. R package version 2.0-1. https://CRAN.R-project.org/package=labdsv.

Tomczak, M & Tomczak, E. (2014). The need to report effect size estimates revisited. An overview of some recommended measures of effect size. Trends in Sport Sciences 1(21), 19-25.

Wang, Q., Garrity, G. M., Tiedje, J. M. and Cole, J. R. (2007). Naïve Bayesian Classifier for Rapid Assignment of rRNA Sequences into the New Bacterial Taxonomy. Appl. Environ. Microbiol. 73, 5261–5267.

Weiss, S., Xu, Z. Z., Peddada, S., Amir, A., Bittinger, K., Gonzalez, A., Lozupone, C., Zaneveld, J. R., Vázquez-Baeza, Y., Birmingham, A., et al. (2017). Normalization and microbial differential abundance strategies depend upon data characteristics. Microbiome 5, 27.

White, T. J., Bruns, T., Lee, S. and Taylor, J. (1990). Amplification and direct sequencing of fungal ribosomal RNA genes for phylogenetics. In PCR-protocols a guide to methods and applications, M. A. Innis, D. H Gelfand, J.J. Sninski, J. J. and T. J. White, eds (Academic Press, 1990). ISBN 9780123721815.
